# Clade-1 Vap virulence proteins of *Rhodococcus equi* are associated with the cell surface and support intracellular growth in macrophages

**DOI:** 10.1371/journal.pone.0316541

**Published:** 2025-01-06

**Authors:** Zeynep Yerlikaya, Raúl Miranda-CasoLuengo, Yuting Yin, Cheng Cheng, Wim G. Meijer

**Affiliations:** 1 UCD School of Biomolecular and Biomedical Science and UCD Conway Institute, University College Dublin, Dublin, Ireland; 2 Department of Microbiology, School of Veterinary Medicine, Firat University, Elazığ, Türkiye; Van Yuzuncu Yil University Faculty of Veterinary Medicine: Yuzuncu Yil Universitesi Veteriner Fakultesi, TÜRKIYE

## Abstract

The multi-host pathogen *Rhodococcus equi* is a parasite of macrophages preventing maturation of the phagolysosome, thus creating a hospitable environment supporting intracellular growth. Virulent *R*. *equi* isolated from foals, pigs and cattle harbor a host-specific virulence plasmid, pVAPA, pVAPB and pVAPN respectively, which encode a family of 17 Vap proteins belonging to seven monophyletic clades. We examined all 17 Vap proteins for their ability to complement intracellular growth of a *R*. *equi ΔvapA* strain, and show that only *vapK1*, *vapK2* and *vapN* support growth in murine macrophages of this strain. We show that only the clade-1 proteins VapA, VapK1, VapK2 and VapN are located on the *R*. *equi* cell surface. The pVAPB plasmid encodes three clade-1 proteins: VapK1, VapK2 and VapB. The latter was not able to support intracellular growth and was not located on the cell surface. We previously showed that the unordered N-terminal VapA sequence is involved in cell surface localisation of VapA. We here show that although the unordered N-terminus of the 17 Vap proteins is highly variable in length and sequence, it is conserved within clades, which is consistent with our observation that the N-terminus of clade-1 Vap proteins plays a role in cell surface localisation.

## Introduction

The actinomycete *Rhodococcus equi* is a facultative intracellular pathogen of phagocytic cells that infects a wide range of animals as well as immunocompromised humans [1, 2]. Virulent *R*. *equi* strains harbor a virulence plasmid containing a pathogenicity island (PAI) that is essential for intracellular growth [3–9]. Interestingly, equine, bovine and porcine isolates harbour different virulence plasmids, each encoding a family of homologous virulence associated (Vap) proteins. This suggests that the virulence plasmids are an important determinant in conferring host tropism, however, the mechanistic basis for this remains unclear [7, 8, 10]. The equine and porcine virulence plasmids are circular with a conserved backbone containing replication and conjugative regions. In contrast, the bovine plasmid is linear and, apart from the PAI, is not related to pVAPA and pVAPB [7, 8].

The virulence plasmid from equine and porcine strains, pVAPA and pVAPB respectively, encode six Vap proteins, whereas the plasmid of the bovine strain, pVAPN, encodes five Vap proteins [7–9]. These seventeen Vap proteins can be assigned to seven monophyletic clades [8]. Although pVapA contains six homologous *vap* genes, only *vapA* is essential and, together with the PAI encoded transcriptional regulators VirR and VirS, is sufficient for intracellular growth [11, 12]. Interestingly, although the pVAB and pVAPN plasmids contain multiple *vap* genes, for these plasmids too only one Vap protein is essential for intracellular growth. The bovine VapN and the porcine VapK1 and VapK2 are required for intracellular growth and are functional equivalents of VapA [7, 13]. The VapK1 and VapK2 sequences differ by just one amino acid, in which glycine at position 60 in VapK1 is replaced by aspartic acid in VapK2 [7]. Interestingly, VapA, VapK1, VapK2 and VapN all belong to clade-1 [8].

Evidence to date shows that *R*. *equi* initially resides in an acidifying vacuole following phagocytosis which induces expression of *vapA* [14]. VapA and VapN cause exclusion of the proton-pumping vacuolar-ATPase complex and neutralise the *R*. *equi* containing vacuole (RCV) by permeabilising the phagosomal and lysosomal membranes. Interestingly, this appears to occur via a novel mechanism that is distinct from pore formation induced by virulence proteins in other pathogenic bacteria [15–17]. The main role of VapA and VapN is therefore to create a hospitable environment facilitating intracellular growth.

The *R*. *equi* cell envelope is complex, consisting of a peptidoglycan–arabinogalactan–mycolic acid matrix forming an outer lipid permeability barrier [18, 19]. VapA is located on the surface of the *R*. *equi* cell envelope, whereas the remaining five Vap proteins encoded by the pVAPA plasmid are secreted into the extracellular environment [20–22]. It remains unclear by which mechanism VapA interacts with the lipid containing cell envelope of *R*. *equi*. We recently showed that the unordered N-terminus of VapA is essential for cell surface localisation and when fused with the core-VapD protein results in cell surface binding of the VapA-VapD hybrid protein. Cell surface localisation of VapA may facilitate incorporation into extracellular vesicles (EV) which could play a role in pathogenesis [22, 23]. The cellular localisation of Vap proteins encoded by pVAPB and pVAPN is unknown.

In this study we interrogated each individual *vap* gene whether it could complement a *R*. *equi vapA* deletion strain. We show that only the clade-1 proteins VapA, VapN, VapK1 and VapK2 support intracellular growth. These are also the only Vap proteins that are associated with the cell surface, which suggests that localisation on the surface of the cell-envelope is important in pathogenesis. Although the unordered N-terminal domain of Vap proteins is not conserved among Vap proteins in general, they are conserved within each Vap clade, suggesting functionality.

## Materials and methods

### Bacterial strains, plasmids and growth conditions

Bacterial strains and plasmids used in this study are listed in Table 1. *E*. *coli* DH5α was grown in lysogeny broth (LB) at 37°C. *R*. *equi* strains were grown at 200 rpm in LB pH5.5 at 37°C. Agar was added for solid media (1.5%, [w/v]). When appropriate, apramycin was added to media at 80 μg/ml (*R*. *equi*) or 50 μg/ml (*E*. *coli*).

**Table 1:** Plasmids and strains used in this study.

Plasmid/Strain	Relevant genotype/description`	Reference
**Plasmids**		
pSET152	Integrative vector carrying *intØC31*, *attP ØC31 site*, *oriT*, *Apr*^*R*^, *lacZα*, *MCSrep*^*Puc*^.	[24]
pVapA	pSET152 containing *vapA* and 679bp upstream of *vapA* containing the *vapA* promoter	[22]
pVapA-ST	pVapA derivative containing a C-terminal Strep-tag.	[22]
pVapD-ST	pSET152 containing a synthetic fusion of VapD under *P*_*vapA*_ containing a C-terminal Strep-tag.	[22]
pVapJ-ST	pSET152 containing a synthetic fusion of VapJ under *P*_*vapA*_ containing a C-terminal Strep-tag.	This study
pVapK1-ST	pSET152 containing a synthetic fusion of VapK1 under *P*_*vapA*_ containing a C-terminal Strep-tag.	This study
pVapK2-ST	pSET152 containing a synthetic fusion of VapK2 under *P*_*vapA*_ containing a C-terminal Strep-tag.	This study
pVapL-ST	pSET152 containing a synthetic fusion of VapL under *P*_*vapA*_ containing a C-terminal Strep-tag.	This study
pVapM-ST	pSET152 containing a synthetic fusion of VapM under *P*_*vapA*_ containing a C-terminal Strep-tag.	This study
pVapN-ST	pSET152 containing a synthetic fusion of VapN under *P*_*vapA*_ containing a C-terminal Strep-tag.	This study
pVapO-ST	pSET152 containing a synthetic fusion of VapO under *P*_*vapA*_ containing a C-terminal Strep-tag.	This study
pVapP-ST	pSET152 containing a synthetic fusion of VapP under *P*_*vapA*_ containing a C-terminal Strep-tag.	This study
pVapR-ST	pSET152 containing a synthetic fusion of VapR under *P*_*vapA*_ containing a C-terminal Strep-tag.	This study
pVapS-ST	pSET152 containing a synthetic fusion of VapS under *P*_*vapA*_ containing a C-terminal Strep-tag.	This study
**Strains**		
*E*. *coli DH5α*	supE44 _ lacU169, (φ80lacZ_ M15) hsdR17 recA1 endA1 gyrA96 thi 1 relA1	Bethesda Research Laboratories
*R*. *equi* 103S	Virulent *R*. *equi* harbouring virulence plasmid pVAPA1037 isolated from horse	[25]
*R*. *equi* 103S^P-^	Plasmid-cured attenuated *R*. *equi* isolate	[26]
*R*. *equi* Δ*vapA*	*R*. *equi* 103S with an unmarked, in-frame, *vapA* deletion.	[26]
*R*. *equi* Δ*vapA/*pVapA	*R*.*equi* Δ*vapA* harbouring pVapA	[22]
*R*. *equi* Δ*vapA/*pVapA-ST	*R*.*equi* Δ*vapA* harbouring pVapA-ST	[22]
*R*. *equi* Δ*vapA/*pVapD-ST	*R*.*equi* Δ*vapA* harbouring pVapD-ST	[22]
*R*. *equi* Δ*vapA/*pVapB-ST	*R*.*equi* Δ*vapA* harbouring pVapB-ST	This study
*R*. *equi* Δ*vapA/*pVapK1-ST	*R*.*equi* Δ*vapA* harbouring pVapK1-ST	This study
*R*. *equi* Δ*vapA/*pVapK2-ST	*R*.*equi* Δ*vapA* harbouring pVapK2-ST	This study
*R*. *equi* Δ*vapA/*pVapL-ST	*R*.*equi* Δ*vapA* harbouring pVapL-ST	This study
*R*. *equi* Δ*vapA/*pVapM-ST	*R*.*equi* Δ*vapA* harbouring pVapM-ST	This study
*R*. *equi* Δ*vapA/*pVapN-ST	*R*.*equi* Δ*vapA* harbouring pVapN-ST	This study
*R*. *equi* Δ*vapA/* pVapO-ST	*R*.*equi* Δ*vapA* harbouring pVapO-ST	This study
*R*. *equi* Δ*vapA/*VapP*-ST*	*R*.*equi* Δ*vapA* harbouring pVapP-ST	This study
*R*. *equi* Δ*vapA/*VapR*-ST*	*R*.*equi* Δ*vapA* harbouring pVapS-ST	This study
*R*. *equi* Δ*vapA/*VapS*-ST*	*R*.*equi* Δ*vapA* harbouring pVapS-ST	This study

### DNA manipulations

All complementation constructs are based on the integrative vector pSET152 [24]. Transcriptional fusions of *vap* genes under the control of the *vapA* promoter were synthesized as gBlocks (Integrated DNA Technologies). The *vapA* promoter is contained within a fragment of 680 bp upstream of the start codon of *vapA* [27]. The gBlocks were flanked with XbaI restriction sites to facilitate cloning in the corresponding site of pSET152. In addition, a sequence encoding the Strep-tag II (WSHPQFEK) was located at the carboxyl-end of the corresponding *vap* genes. Plasmids were purified from *E*. *coli* DH5α using the High pure Plasmid Isolation Kit (Roche). These were introduced into *R*. *equi* 103S Δ*vapA*, a derivative strain harbouring an in frame deletion of *vapA* [26] by electroporation using a GenePulser II coupled to a Pulse Controller Plus (BioRad) as previously described [28]. The genotype of the resulting strains was verified by PCR (S1 Fig) using oligonucleotides specified in S1 Table.

### Reverse transcription

Total RNA isolated using the RNeasy Mini Kit (Qiagen) was used as template for reverse transcription with the Improm II reverse transcriptase (Promega) and random 6-mer primers (Thermo-Fisher) followed by PCR with *vap* specific oligonucleotides (Table 2) using KAPA2G Fast HotStart DNA Polymerase (Sigma Aldrich).

**Table 2:** Oligonucleotides used for RT-PCR.

Oligonucleotide	Sequence (5’ -> 3’)	Reference
vapB-196F	GAGGTCGGTTCTCAGGCATA	This study
vapB-196R	CGTCACCATCGAAGACCATA	This study
VapJ-232f	CTGCCCCATTCCAGTTTTCC	This study
VapJ-232r	AATTGCTCGCCTCCATCAAC	This study
VapK-170f	AGTCATGGTTCCTGCCGG	This study
VapK-170r	AGCCGCTACCGTCGTAATC	This study
VapL-156f	GAACGGGCAGCTATCTTTCG	This study
VapL-156r	GAAATCCCGCCAGAATCACC	This study
VapM-159f	GGGGAGTTTGCATCTGTGTC	This study
VapM-159r	ATCGCCATCAAAAGTCTCGC	This study
VapN-170F	GACGTCAAGGGCAACGTTAT	This study
VapN-170R	GCGGTATCGGAGTAGAGACG	This study
VapO_183F	GCGAACGCAGAAACTCCTAC	This study
VapO_183R	GACAAGACCGTGAACCGAAT	This study
VapR_230F	GACTCGGAGCAGCAGTATCC	This study
VapR_230R	CCGACAGCGTTGTACTCAAA	This study
VapS_220F	GCACTGTACGTGCCGAAGTA	This study
VapS_220R	TCTCCATCGAAGGTCATTCC	This study

### Flow cytometry

*R*. *equi* grown overnight in LB medium was harvested, washed twice in PBS and their concentration adjusted to OD_600_ of 1.0 equivalent to 1.5 x 10^8^ cells ml^-1^. Aliquots of 5μl containing 7.5 x 10^5^ cells were transferred to 1.5 ml tubes and fluorescently labelled with an anti-Strep-Tag II α-Strep tag FITC-mAb (GenScript). Briefly, 100 μl of a 0.5 μg/ml solution of the α-Strep tag FITC-mAb was added and incubated in the dark for 30 min at room temperature. Subsequently, 200 μl propidium iodide (PI, Biosciences, United Kingdom) was added to a final concentration of 78 ng/μl just before injecting into a CytoFLEX LX flow cytometer (Beckman Coulter). The fluorescence of FITC, PI and scattered light of 50,000 cells (or 2 minutes of recording, whichever occurred first) were simultaneously recorded. Heat-inactivated and unstained *R*. *equi* controls were used to set up the gates for the visualization of four different single cell populations. Live FITC-unstained cells, live FITC-stained cells, dead FITC-unstained cells and dead FITC-stained cells. The gating strategy employed to detect the population of alive FITC-labeled cells was previously described [22]. *R*. *equi* surface proteins were removed by proteolysis through incubation of cells with trypsin (0.05% [w/v]) for 30 min at room temperature. At least three biological replicates were performed for each condition and strain. The Stain Index (SI) was used to assess the brightness of fluorescence signal in live FITC-stained cells relative to Live FITC-unstained [29].

### Macrophage infections

*R*. *equi* strains grown overnight on LB broth were harvested by centrifugation for 10 min at 3000 x *g* and washed twice in PBS free of Ca^2+^ and Mg^2+^. Murine macrophage-like cells J774A.1 (American Type Culture Collection) were cultured overnight in Dulbecco’s modified Eagle’s medium (DMEM) supplemented with 10% (v/v) foetal bovine serum and 2 mM L-glutamine and incubated at 37°C in 5% CO_2_. Cells were the washed in pre-warmed phagocytosis buffer [0.1% (w/v) gelatine, equal amounts of Medium 199 and DMEM and suspended in the phagocytosis buffer supplemented with 5% mouse serum. 6x10^5^ cells/ml (final volume 1 ml) were seeded in 24 well plates (Sarstedt) and grown overnight at 37°C in 5% CO_2_. Cells were infected with *R*. *equi* at a multiplicity of infection (MOI) of 10. Infections were started by centrifugation (160x*g* for 3 min) to synchronize bacterial internalization. Plates were incubated for 45 min at 37°C in 5% CO_2_ and the monolayers were washed three times in pre-warmed phagocytosis buffer (37°C) to remove unbound bacteria. A further incubation of 15 min allowed the internalization. Monolayers were washed again with warm phagocytosis buffer and subsequently suspended in DMEM supplemented with 10% (v/v) foetal calf serum, 4 mM L-glutamine, 1% non-essential amino acids and 10 μg/ml amikacin (this time point was denominated t = 0). Infected monolayers were harvested at different time points between 4 and 48 h. Medium was replaced after 24 h with fresh medium containing 10 μg/ml amikacin. Intracellular proliferation of *R*. *equi* was monitored by determining the fold change of 16S rRNA per monolayer using qPCR in a LightCycler II (Roche) as previously described [26]. All experiments were conducted in triplicate.

### Phylogenetic analysis

A multiple sequence alignment was generated using Clustal X v.2.1 [30] using mature Vap proteins derived from pVAPA1037 (NC_011151.1), pVAPB1593 (NC_011150.1) and pVAPN1571 (NZ_KF439868.1) plasmid sequences. The position of the signal peptidase II cleavage site was predicted using SignalP6.0 [31] and was used to remove the signal sequence to generate the mature protein sequence. The identification of the unordered N-terminal Vap domains is based on the secondary and tertiary structural analysis of VapB, VapD and VapG proteins, which identified the highly conserved and tightly folded core Vap domain [32–34]. Maximum-likelihood phylogenetic analysis of mature Vap proteins was carried out using PhyML v3.0 [35].

### Data analysis

Statistical analysis was performed in GraphPad Prism version 10. Flow cytometry data were log2 transformed for SI analysis.

## Results

### Only clade-1 Vap proteins support intracellular growth of *R*. *equi*

It has been shown previously that *vapK1* and *vapK2* can restore intracellular growth of *R*. *equi ΔvapA* whereas deletion of *vapN* abolishes intracellular growth, showing that the porcine *vapK1* and *vapK*2 and bovine *vapN* are functional equivalents of the equine *vapA* [8, 13]. The equine pVAPA plasmid contains five *vap* gene in addition to *vapA*, none of which were able to complement *R*. *equi ΔvapA* nor were any of the equine Vap proteins attached to the *R*. *equi* cell surface [22]. Cell surface localisation and the ability to support intracellular growth may therefore be key characteristics of clade-1 Vap proteins.

**Fig 1:**
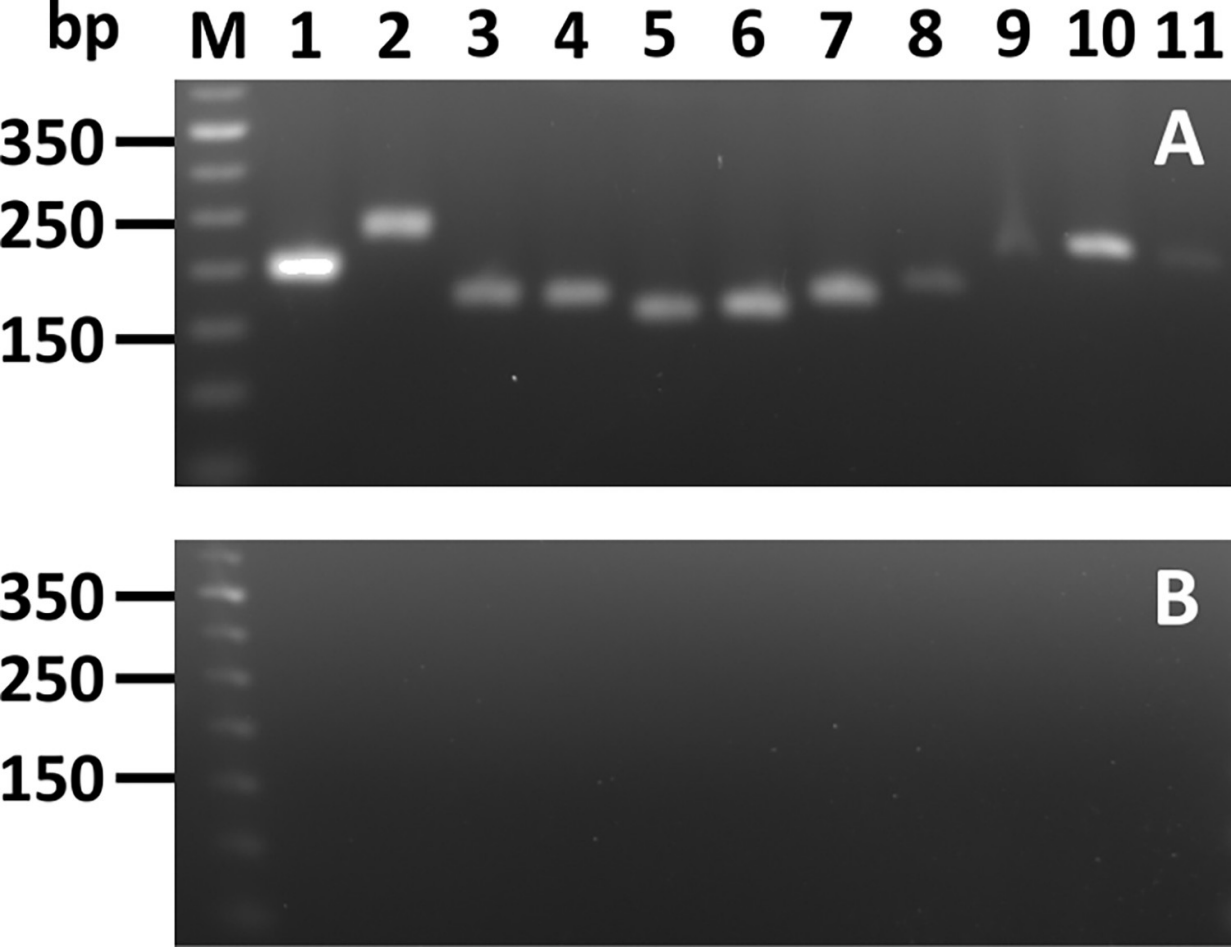
Qualitative assessment transcriptional expression of *vap*-ST genes in *R*. *equi* 103S Δ*vapA*. A) Total RNA isolated from the relevant strain was used as template for reverse transcription with the Improm II reverse transcriptase and random 6-mer primers followed by PCR with KAPA2G Fast DNA. For the PCR step, a common forward primer (PvapA_screenF) targeting a sequence within the intergenic sequence containing the *vapA* promoter was employed together with Vap-specific reverse primers (Table 2) as required for each strain. Lanes: 1) pVapB-ST (196 bp), 2) pVapJ-ST (232 bp), 3) pVapK1-ST (170 bp), 4) pVapK2-ST (170 bp), 5) pVapL-ST (156 bp), 6) pVapM-ST (159 bp), 7) pVapN-ST (170 bp), 8) pVapO-ST (183 bp), 9) VapP-ST (216 bp) 10) VapR-ST (230 bp), 11) VapS-ST (220 bp) and M) DNA ladder (Invitrogen). B) Non-reverse transcriptase control.

To examine this hypothesis in detail, we embarked on a systematic analysis of *vap* genes of the pVAPB and pVAPN plasmids to determine whether these can complement *R*. *equi ΔvapA* and whether their products are located on the cell surface. To this end we generated a set of genome-integrative constructs in which individual *vap* genes containing a sequence encoding a carboxy-terminal Strep-tag II (*vap-ST*) are expressed from the *vapA* promoter (Table 1). Expression of each *vap-ST* was corroborated at transcriptional level using RT-PCR (Fig 1).

**Fig 2:**
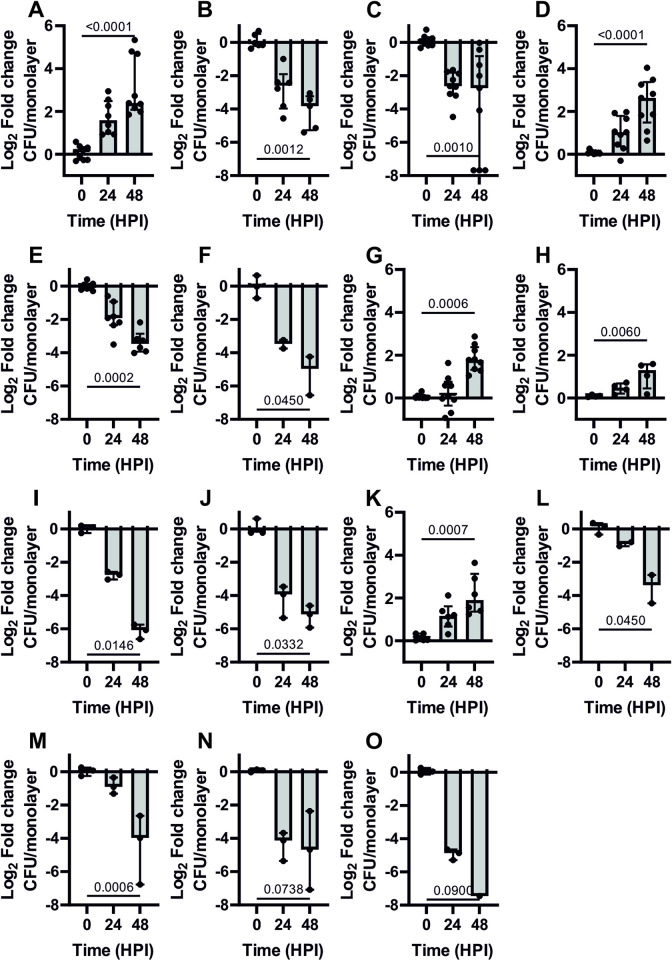
Complementation of the loss of intracellular replication of *R*. *equi* 103S Δ*vapA* with carboxyl-Strep-tag Vaps encoded in the porcine- and the bovine-type virulence plasmids. J774A.1 macrophage-like cells were infected with virulent *R*. *equi* 103S (A), plasmid-cured, *R*. *equi* 103S^P-^ (B) and *R*. *equi* 103S Δ*vapA*, an attenuated derivative harbouring an *in-frame*, unmarked, deletion of *vapA* (C). The latter was used for complementation analysis using pVapA-ST (*R*. *equi* Δ*vapA/*pVapA-ST) (D) as a positive control and other carboxyl-Strep Tagged *vaps* from the porcine-type pVAPB1593 (E-J) and bovine-type pVAPN1571 (K-O) virulence plasmids. Infections were set at a MOI of 10 as described in Materials and Methods and the intracellular replication assessed at 24 and 48 hours post infection (HPI) an reported as the Log2 Fold change of CFU per monolayer as compared with time zero. E) *R*. *equi* Δ*vapA/*pVapB-ST; F) *R*. *equi* Δ*vapA/*pVapJ-ST; G) *R*. *equi* Δ*vapA/*pVapK1-ST; H) *R*. *equi* Δ*vapA/*pVapK2-ST; I) *R*. *equi* Δ*vapA/*pVapL-ST; J) *R*. *equi* Δ*vapA/*pVapM-ST; K) *R*. *equi* Δ*vapA/*pVapN-ST; L) *R*. *equi* Δ*vapA/*pVapO-ST; M) *R*. *equi* Δ*vapA/*pVapP-ST; N) *R*. *equi* Δ*vapA/*pVapR-ST; O) *R*. *equi* Δ*vapA/*pVapS-ST. Bars represent the median of at least three independent experiments measured in triplicate. Error bars represent the interquartile range. In some cases, bacteria was cleared during the time course of the experiment. In these cases, data shown only represents values that were possible to count. Statistical analysis was performed using a Kruskal-Wallis test. P-values were adjusted using the Dunn’s correction for multiple comparisons.

Infection of J774A.1 macrophage-like cells with the virulent strain *R*. *equi* 103S, resulted in an increase of intracellular bacteria over the course of the infection with median fold changes of colony forming units (CFU) per monolayer relative to time zero of 3.01 (interquartile range (IQR) 1.94–5.58) and 5.22 (IQR 4.22–26.80) at 24 and 48 hours post infection (HPI), respectively (Fig 2A). In contrast, the fold change of the avirulent strains *R*. *equi* 103S^P-^, lacking the virulence plasmid, and *R*. *equi* Δ*vapA*, significantly decreased over the same timeframe (Fig 2B and 2C). Complementation of *R*. *equi* Δ*vapA* with pVapA-ST restored intracellular replication with a median fold change of 6.19 (IQR 2.79 to 10.32) at 48 HPI (Fig 2D). In contrast, pVapB-ST, from the pVAPB virulence plasmid, failed to complement the loss of *vapA* as observed by a statistically significant reduction in CFU/monolayer fold change (Fig 2E). However, the fold changes of bacteria transformed with either pVapK1-ST and pVapK2-ST significantly increased over the course of the infection with fold changes at 48 HPI of 3.42 (IQR 2.49–5.17) and 2.46 (IQR 1.36–2.97), respectively (Fig 2G and 2H). Complementation of *R*. *equi* Δ*vapA* with pVapN-ST produced median fold changes of 3.72 (IQR 2.57–8.75) after 48 hours (Fig 2F). In contrast, all other *vap* genes from either pVAPB (Fig 2F, 2I and 2L) and pVAPN (Fig 2N, 2I and 2L) virulence plasmids failed to complement the loss of *vapA* in *R*. *equi* Δ*vapA*. These data unequivocally show that only clade-1 Vap proteins are capable of supporting intracellular growth in macrophages. Interestingly, pVAPB encodes three clade-1 Vap proteins, however, only two, VapK1 and VapK2 are functionally homologous to VapA.

### Surface localization is a unique characteristic of clade-1 Vap proteins

**Fig 3:**
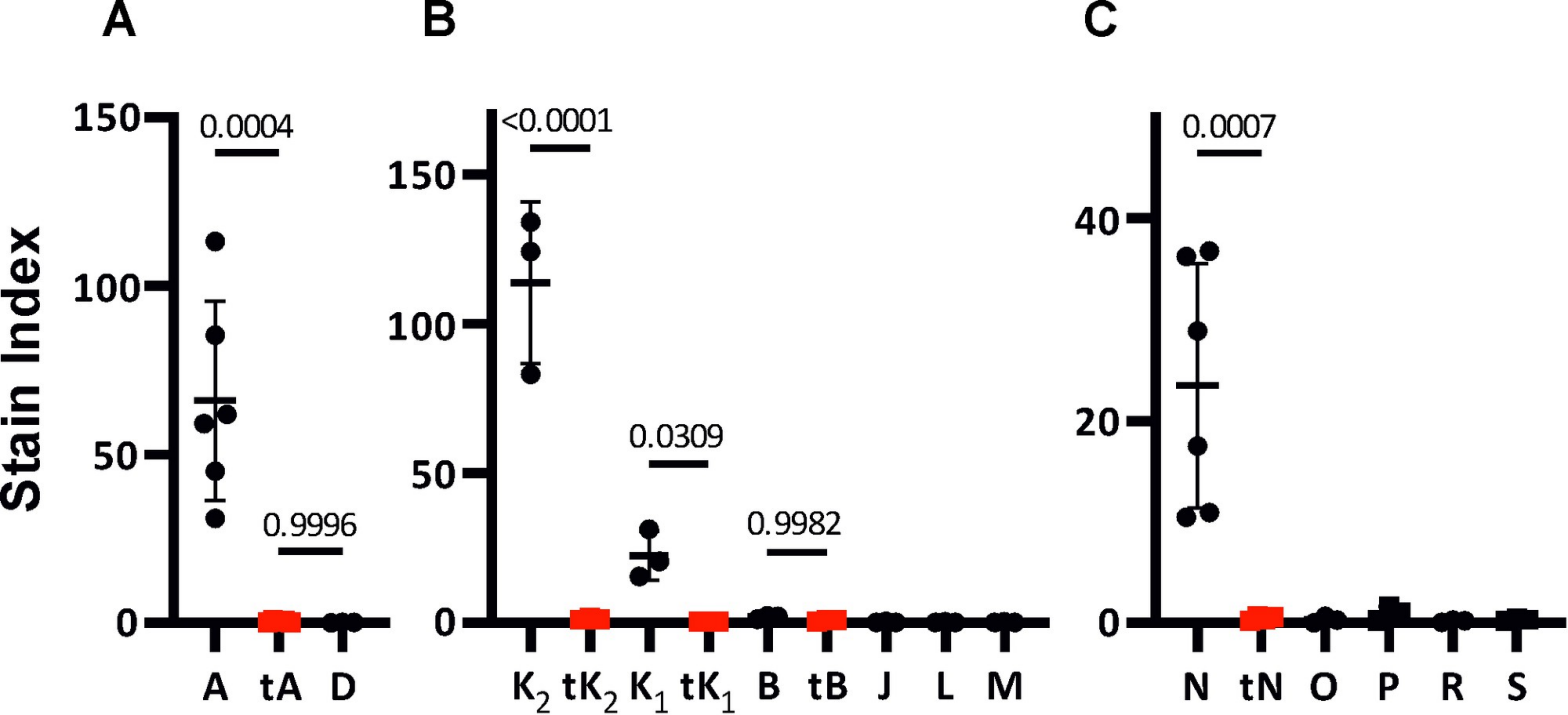
Only clade-1 Vap proteins are localised on the cell surface of *R*. *equi*. *R*. *equi* 103S Δ*vapA* transformed with plasmids encoding carboxyl-terminal Strep tagged derivatives of relevant Vaps were stained with the α-Strep tag FITC-mAb and analysed by Flow cytometry. Alive, FITC-stained singlet cells were measured as described in Materials and Methods. The stain index was used to quantify the brightness of stained (positive) cells relative to their unstained (negative) controls. Panels show the log_2_ results obtained for Vaps encoded by A) the equine type pVAP1037 virulence plasmid, B) the porcine type pVAPB1593 virulence plasmid and C) bovine type pVAPN1571 virulence plasmid (Black symbols). Trypsin digestion before incubation with the α-Strep tag FITC-mAb was employed to confirm the exposure of Strep-tagged proteins to the extracellular environment (Red symbols). Only selected Vaps encoded in the equine type virulence plasmid are shown as example of a protein expressed on the cell surface (VapA-ST) and a protein that is not (VapD-ST), as previously reported. Dot plots of at least three independent experiments are shown. Error bars represent the mean and standard deviation. Statistical analysis was performed in GraphPad using one-way ANOVA. Correction for multiple comparisons was performed using the Šídák test. The p-value of relevant comparisons is depicted above horizontal lines.

We previously showed that out of the six pVAPA vap proteins, only the clade-1 protein VapA is located on the bacterial cell surface [22]. Since only clade-1 Vap proteins are capable of supporting intracellular growth, we hypothesized that cell surface localisation is an important characteristic of clade-1 Vap proteins. To test this hypothesis, intact *R*. *equi* cells expressing individual *vap*-*ST* genes (Table 1) were labelled with α-Strep-tag FITC-mAb to detect surface localisation of Vap proteins. Trypsin digestion before incubation with the α-Strep tag FITC-mAb was employed to confirm the exposure of Strep-tagged proteins to the extracellular environment. The Stain Index (SI) was measured to determine the brightness of a sample relative to their unstained controls (Fig 3).

*R*. *equi* 103S Δ*vapA*/pVapA-ST produced high fluorescence signal with a stain index (SI) of 65.96 ± 29.46 brighter than its unstained control (Fig 3A). The cell surface localization was confirmed by the loss of the binding sites on cells that were digested with trypsin before incubation with the α-Strep tag FITC-mAb, which resulted in a significantly lower SI of 0.52 ± 0.32 (Fig 3A) that was not different than the recorded for bacteria transformed with pVapD-ST (Fig 3A). *R*. *equi* 103S Δ*vapA*/VapK1-ST and *R*. *equi* 103S Δ*vapA*/VapK2-ST produced SI values of 113.76 ± 27.22 and 22.35 ± 8.16. Cells expressing the bovine VapN-ST protein produced high SI values (23.47 ± 12.09). The SI values of cells expressing these clade-1 Vap proteins decreased by two orders of magnitude following digestion with trypsin (0.49 ± 0.21). The SI of *R*. *equi* 103S Δ*vapA/pVap*B-ST was very low and did not significantly (P>0.05) decrease following treatment with trypsin (Fig 3B), which demonstrates that VapB-ST protein does not accumulate on the cell surface of *R*. *equi*. The remaining pVAPB Vap-ST proteins (VapJ-ST, VapL-ST, VApM-ST) and pVAPN proteins (VapO-ST, P-ST, R-ST and S-ST) also produced very low SI values (Fig 3C). These data thus show that surface binding is restricted to clade-1 Vap proteins.

### Conservation of the unordered N-terminal Vap domain is clade specific

**Fig 4:**
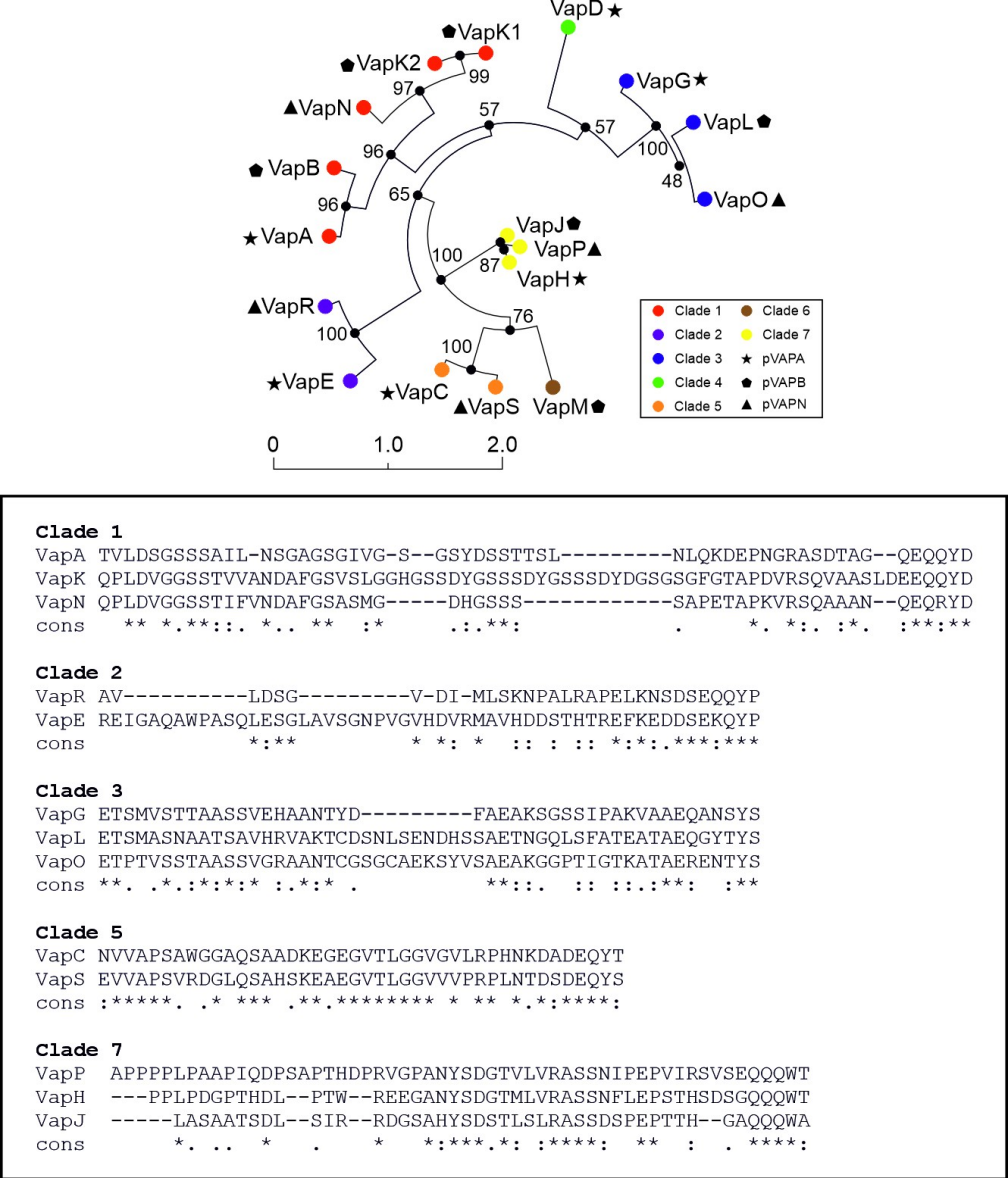
Conservation of the unordered N-terminal Vap domain is clade specific. A) Unrooted maximum likelihood tree of the Vap protein family encoded by pVAPA (VapA, VapD, VapG, VapH, VapE), pVAPB (VapB, VapK1, VapK2, VapJ, VapL, VapM) and pVAPN (VapN, VapO, VapP, VapR, VapS). The ML tree is based on an alignment of mature Vap proteins, excluding the signal sequence. The number of bootstraps (n = 100) are indicated at the branch points. B) Sequence alignment of the unordered N-terminal sequences of clade-1, clade-2, clade-3, clade-5 and clade-7 proteins. The unordered N-terminal Vap sequences are derived from mature Vap proteins excluding the core Vap proteins as indicated by the structural analysis of VapB, VapD and VapG proteins [32–34]. The N-terminal sequences of VapK1 and VapK2 are identical and only included once in the sequence alignment. The VapB N-terminal sequence is excluded from the alignment because it is not a functional VapA homologue, unlike the other clade-1 proteins.

We recently showed that the N-terminal domain of VapA appears to be an important determinant in cell-surface localisation of this protein [22]. This is remarkable, given that in contrast to the highly conserved core-Vap protein, the unordered N-terminal domain is not conserved when considering all 17 Vap proteins. The lack of sequence conservation seems to argue against a functional role for this domain. However, inspection of each clade individually reveals that the N-terminal domain is conserved within a clade. Three of the seven Vap clades contain representatives from pVAPA, pVAPB and pVAPN (Fig 4A). The N-terminal domains of clades 1, 3 and 7 show a high degree of sequence similarity within their clades, despite the fact that each of these are encoded by virulence plasmids of *R*. *equi* strains with a different tropism. The N-terminal domains of clade-1 proteins are characterised by a high glycine and serine content, which distinguishes these from other Vap N-termini (Fig 4B).

## Discussion

A hallmark of the PAI of the *R*. *equi* virulence plasmids is the presence of multiple *vap* genes, which appear to have arisen through gene duplication events from a single ancestral gene [8]. To date three types of *R*. *equi* virulence plasmids have been identified, which encode a total of 17 Vap proteins that belong to seven monophyletic clades. Interestingly only clade-1 proteins are necessary for intracellular growth, the function of the remaining Vap proteins remains enigmatic. We previously determined that of the six equine Vap proteins, only VapA, which is required for intracellular growth, is located on the cell surface of *R*. *equi* [22]. We therefore hypothesized that cell surface localisation is a hallmark of clade-1 Vap proteins.

In this study we interrogated all 17 Vap proteins whether these can complement a *R*. *equi* Δ*vapA* strain, and showed that only clade-1 Vap proteins, VapA, VapK1 VapK2 and VapN, can do so. The fact that bovine and porcine Vap proteins restored intracellular replication of an equine *R*. *equi* Δ*vapA* strain demonstrates that the mechanism for Vap secretion and cell surface anchoring is not dependent on the *R*. *equi* genetic background. This is consistent with the observation that replication in equine, bovine and porcine macrophages is not strain dependent [10], which strongly suggests that clade-1 Vap proteins are not critical in determining host tropism.

Although the three types of virulence plasmids encode 17 Vap proteins, cell surface localisation is unique to clade-1 Vap proteins. Considering that only clade-1 Vap proteins support intracellular growth it appears likely that attachment to the cell envelope is an important characteristic of this class of virulence factors. It was recently shown that *R*. *equi* produces extracellular vesicles containing VapA that mediate a macrophage inflammatory response via the TLR2-NF-κB/MAPK pathways [23]. The localisation of clade-1 Vap proteins on the cell envelope may therefore facilitate inclusion in extracellular vesicles produced by *R*. *equi* and could play a key role in host-pathogen interaction [23].

Within phagocytic cells VapA is present on the bacterial surface and at the membrane of RCVs, suggesting the protein has an affinity for lipids [15, 36]. This was confirmed by the observation that VapA specifically interacts with liposomes containing phosphatidic acid [36]. VapN, the functional homologue of VapA also interacts with host membranes [17]. The mechanism underlying the interaction between clade-1 Vap proteins and the bacterial cell surface is unknown. Structural analysis of VapB, VapD and VapG showed that Vap proteins are characterised by a variable, disordered N-terminal domain, followed by a highly conserved core that folds into an antiparallel β-barrel formed by eight β-strands separated in the middle by a short α-helix [32–34]. Core-VapA retains its ability to permeabilise the membrane of the RCV preventing its acidification, showing that the unordered N-terminal domain of VapA is not required for intracellular replication [15, 37]. The unordered N-terminal domain of the 17 Vap proteins identified to date differ considerably in both length and sequence. However, closer inspection revealed that the N-terminal domain is conserved within the clade the Vap protein belongs to, suggesting functionality. This was confirmed using a VapA-VapD hybrid protein. VapD is not associated with the cell surface and has a short N-terminal sequence. However, a VapA-VapD hybrid, combining the disordered VapA N-terminal sequence with the VapD core protein, is located on the cell surface [22].

In contrast to the pVAPA and pVAPN virulence plasmids, the porcine pVAPB plasmid encodes not one but three clade-1 Vap proteins, VapK1, VapK2 and VapB. The VapK1 and VapK2 proteins differ by a single amino acid and are clearly the result of a recent gene duplication event. VapK1 and VapK2 restore intracellular growth of *R*. *equi ΔvapA*. Furthermore, like VapA and VapN, these proteins are located on the cell surface. In sharp contrast, *vapB* does not complement *R*. *equi ΔvapA* nor is the VapB protein located on the cell surface. It was noted that porcine and human *R*. *equi* isolates express a surface located 20 kDa protein that is antigenically cross-reactive with VapA antibodies [6, 38]. Prior to a functional analysis of VapB it was generally assumed that this 20kDa surface protein is the product of the *vapB* gene located on the pVAPB plasmid [39]. However, *vapB* does not complement *R*. *equi ΔvapA*, which is confirmed in this study. Furthermore, in contrast to VapK1 and VapK2, VapB is not located on the cell surface. The 20kDa surface protein of virulent human and porcine *R*. *equi* strains which was assumed to be VapB, is therefore most likely VapK1 and VapK2.

The fact that only clade-1 Vap proteins are required for growth in macrophages raises the question what the function of the other Vap proteins is. It is a distinct possibility that Vap proteins associated with the other clades are required for growth in niches other than mammalian macrophages, for example within predatory protozoa or other as yet unidentified hosts. For example, *Mycobacterium* species infect a diverse range of hosts in addition to mammals, including fish, insects and amoeba [40–44]. Interestingly, it was recently shown that uptake of pVAPA cured *R*. *equi* ATCC33701 by *Acanthamoeba castellanii* is significantly higher than strains containing pVAPA. In contrast, pVAPA containing *R*. *equi* showed significantly better intracellular growth, suggesting the pVAPA virulence plasmid provides a defence against predation by amoeba [45]. It is not know which of the six pVAPA encoded Vap proteins play a role in enhancing intracellular growth in *A*. *castellanii*.

All three types of virulence plasmids contain *vap* pseudogenes that are no longer functional. Interestingly, except for clade-1, no clade contains more than one functional Vap protein for each of the three types of virulence plasmids. These pseudogenes could have arisen because the niche for which their functional precursor was required is no longer occupied by the pathogen, allowing mutations to accumulate. Alternatively, gene duplication gives rise to redundancy such that genetic drift of a redundant gene allows adaption to a different role. Clade-1 contains three pVAPB encoded *vap* genes, two of which, *vapK1* and *vapK2*, are functionally homologous to VapA and VapN. The fact that, except for clade-1, no clade contains more than one functional Vap protein for each of the three types of virulence plasmids, suggests that, although *vapB* is expressed, it has already been subject to loss of function and is likely to eventually degenerate into a pseudo-gene. In this scenario, either of the two *vapK* genes may also become subject to genetic drift and become inactive.

## Conclusion

To date three types of virulence plasmids have been identified, encoding 17 Vap proteins belonging to seven monophyletic clades [8] (Fig 4B). Only the clade-1 Vap proteins VapA, VapK1, VapK2 and VapN support intracellular growth in mammalian macrophages and, in addition, are the only Vap proteins associated with the *R*. *equi* cell surface. We previously showed that the unordered VapA N-terminal sequence plays a role in cell surface localisation. Interestingly, the N-terminal sequences, although not conserved in general, are conserved within clades, suggesting functionality. Cell surface localisation may serve to facilitate inclusion in extracellular vesicles produced by *R*. *equi*.

## Supporting information

S1 Fig Genotyping of *R*. *equi* 103S Δ*vapA* and strain derivatives. (PDF)

S1 Raw image This is the raw image for Fig 1. (PDF)

S2 Raw image These are the raw images for S1 Fig. (PDF)

S1 Table Oligonucleotides used for genotyping of *R*. *equi* 103S Δ*vapA* and strain derivatives. (PDF)

S1 File This an excel file containing the data for Fig 2. (XLSX)

S2 File This is an excel file containing the data for Fig 3. (XLSX)
