# Supplementary material for: Clade-1 Vap virulence proteins of Rhodococcus equi are associated with the cell surface and support intracellular growth in macrophages
Source: PLoS One. 2025 Jan 6;20(1):e0316541. doi: 10.1371/journal.pone.0316541 (PMC11703076; doi:10.1371/journal.pone.0316541)
Supplement: S1 Fig — (PDF) [file pone.0316541.s001.pdf]

Clade-1 Vap virulence proteins of *Rhodococcus equi* are associated with the cell surface and support intracellular growth in macrophages

Supporting Information

Zeynep Yerlikaya[1,2], Raúl Miranda-CasoLuengo[1], Yuting Yin[1], Cheng Cheng[1]

and Wim G. Meijer[1*]

[1] UCD School of Biomolecular and Biomedical Science and UCD Conway Institute, University College Dublin, Dublin 4, Ireland.
[2] Department of Microbiology, School of Veterinary Medicine, Firat University, Elazığ, Türkiye

*Corresponding author

E-mail: wim.meijer@ucd.ie

Keywords: Targeting; surface protein; fusion protein; intracellular growth; pathogen; flow cytometry

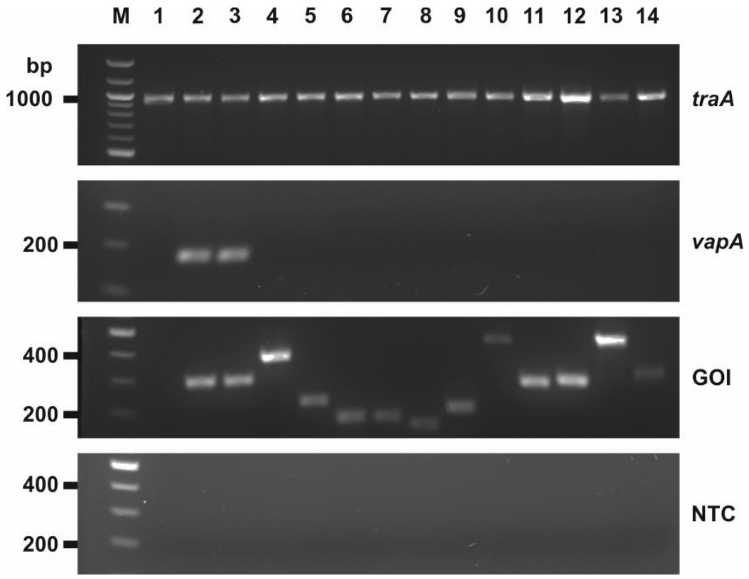

S1 Figure: Genotyping of *R. equi* 103S Δ*vapA* and strain derivatives. Each strain was genotyped by PCR amplification using specific primers (see S1 Table) against the following targets. A) *traA* (960 bp), B) vapA (182 bp), C) gene of interest (GOI) depending on the strain (see Lanes below) and D) Non-template control (NTC) against *vap* of interest. The *traA* amplicon shows the presence of the virulence plasmid, the *vapA* amplicon the presence of *vapA*. The GOI, gene of interest, represents the *vap* gene cloned into pSET152 and is integrated into the chromosome. Boiled preparations of single colonies were used as template. Each lane corresponds to specific strains as follows: 1) *R. equi* 103S Δ*vapA*, 2) pVapA (336 bp), 3) pVapA-ST (336 bp), 4) pVapB-ST (422 bp), 5) pVapJ-ST (258 bp), 6) pVapK1-ST (210 bp), 7) pVapK2-ST (210 bp), 8) pVapL-ST (189 bp), 9) pVapM-ST (234 bp), 10) pVapN-ST (481 bp), 11) pVapO-ST (318 bp), 12) pVapP-ST (328 bp), 14) pVapR-ST (501 bp), 15) pVapS-ST (360 bp) and M) DNA ladder. Quick-Load® Purple 100 bp DNA Ladder (NEB Biolabs).
