# Supplementary material for: Clade-1 Vap virulence proteins of Rhodococcus equi are associated with the cell surface and support intracellular growth in macrophages
Source: PLoS One. 2025 Jan 6;20(1):e0316541. doi: 10.1371/journal.pone.0316541 (PMC11703076; doi:10.1371/journal.pone.0316541)
Supplement: S1 Raw image — (PDF) [file pone.0316541.s002.pdf]

Clade-1 Vap virulence proteins of *Rhodococcus equi* are associated with the cell surface and support intracellular growth in macrophages

Supporting Information

Zeynep Yerlikaya[1,2], Raúl Miranda-CasoLuengo[1], Yuting Yin[1], Cheng Cheng[1]

and Wim G. Meijer[1*]

[1] UCD School of Biomolecular and Biomedical Science and UCD Conway Institute, University College Dublin, Dublin 4, Ireland.
[2] Department of Microbiology, School of Veterinary Medicine, Firat University, Elazığ, Türkiye

*Corresponding author

E-mail: wim.meijer@ucd.ie

Keywords: Targeting; surface protein; fusion protein; intracellular growth; pathogen; flow cytometry

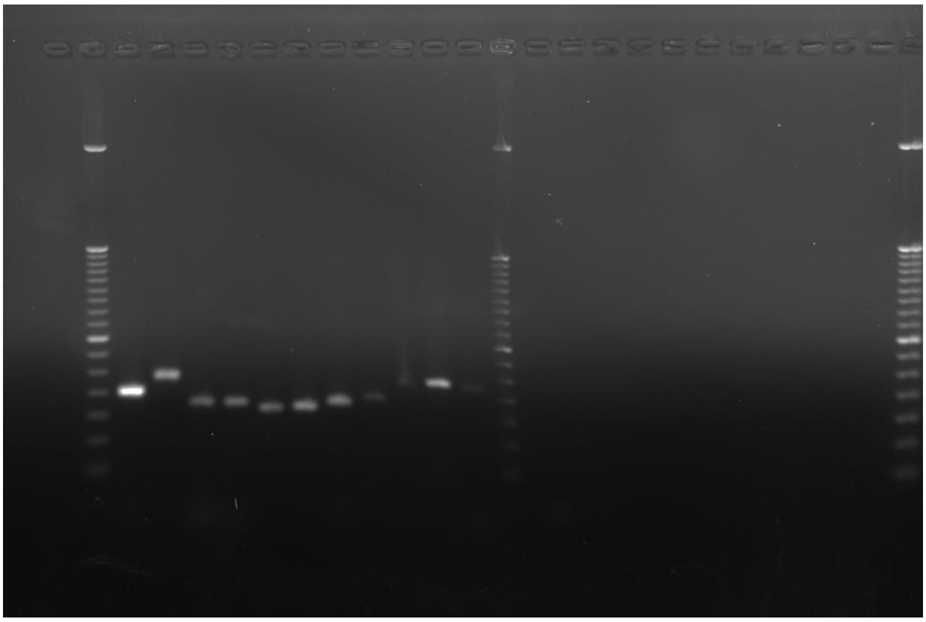
Raw gel data used in Figure 1.
