# Supplementary material for: Clade-1 Vap virulence proteins of Rhodococcus equi are associated with the cell surface and support intracellular growth in macrophages
Source: PLoS One. 2025 Jan 6;20(1):e0316541. doi: 10.1371/journal.pone.0316541 (PMC11703076; doi:10.1371/journal.pone.0316541)
Supplement: S1 Table — (PDF) [file pone.0316541.s004.pdf]

# Clade-1 Vap virulence proteins of *Rhodococcus equi* are associated with the cell surface and support intracellular growth in macrophages

Supporting Information

Zeynep Yerlikaya[1,2], Raúl Miranda-CasoLuengo[1], Yuting Yin[1], Cheng Cheng[1] and Wim G. Meijer[1*]

[1] UCD School of Biomolecular and Biomedical Science and UCD Conway Institute, University College Dublin, Dublin 4, Ireland.
[2] Department of Microbiology, School of Veterinary Medicine, Firat University, Elazığ, Türkiye

*Corresponding author

E-mail: wim.meijer@ucd.ie

Keywords: Targeting; surface protein; fusion protein; intracellular growth; pathogen; flow cytometry

**S1 Table: Oligonucleotides used for genotyping of *R. equi* 103S ΔvapA and strain derivatives**

| Oligonucleotide | Sequence (5' -> 3') | Reference |
|---|---|---|
| TraA_960F | AGAGTTCATGCGTGACAACG | [1] |
| TraA_960R | GTCCACAGGTCACCGTTCTT | [1] |
| VapA_182F[a] | TCTCCGTGAACGTCGTACTG | This study |
| PvapA_screenF[b] | GGAACTGCCCGAGAACATAG | This study |
| VapA_336R[a,b] | TCTCCGTGAACGTCGTACTG | This study |
| VapB_422R[b] | CGTCACCATCGAAGACCATA | This study |
| VapJ_258R[b] | TCAGAAGAAGCACGCAAAGA | This study |
| VapK_210R[b] | AAAGCGTCATTCGCTACGAC | This study |
| VapL_189R[b] | TTGGATGCCATTGATGTTTC | This study |
| VapM_234R[b] | TCCGACACAGATGCAAACTC | This study |
| VapN_481R[b] | GCGGTATCGGAGTAGAGACG | This study |
| VapO_318R[b] | GACAAGACCGTGAACCGAAT | This study |
| VapP_328R[b] | CACTGTCCACTGTTGCTGCT | This study |
| VapR_501R[b] | CCGACAGCGTTGTACTCAAA | This study |
| VapS_360R[b] | TCTCCATCGAAGGTCATTCC | This study |

[a] Forward and reverse primers used for *vapA*. Amplification product (182 bp).

[b] Reverse primer used together with the PvapA_screenF forward primer. The number in the right part of the primer name corresponds to the length of the amplification product (bp).

## References


1. Ocampo-Sosa AA, Lewis DA, Navas J, Quigley F, Callejo R, Scortti M, et al. Molecular epidemiology of *Rhodococcus equi* based on *traA*, *vapA*, and *vapB* virulence plasmid markers. J Infect Dis. 2007;196(5):763-9.
